# Mosaic Fleur-de-Profunda Artery Perforator Flap for Autologous Breast Reconstruction

**DOI:** 10.1097/GOX.0000000000002166

**Published:** 2019-03-11

**Authors:** Lynn Bourn, Radbeh Torabi, Mark W. Stalder, Hugo St. Hilaire, Robert J. Allen, Stephen J. Delatte, Tim S. Matatov, Rozbeh Torabi, Joseph Zakhary, Oren Tessler

**Affiliations:** From the * School of Medicine, Louisiana State University Health Sciences Center, New Orleans, La.; † Section of Plastic & Reconstructive Surgery, Department of Surgery, Louisiana State University Health Sciences Center, New Orleans, La.; ‡ Breast Cancer Specialists of Louisiana, Lafayette, La.; § Elite Plastic Surgery, Phoenix, Ariz.

## Abstract

Perforator-free flaps, in autologous breast reconstruction, have expanded to exploit tissue available at smaller donor sites while retaining high success and low risk rates. Abdominal based flaps, such as the deep inferior epigastric perforator, remain the most common; however, when the abdomen is not an appropriate donor site, lower extremity flaps are options. The profunda artery perforator has the benefit of hiding unsightly scar in the gluteal crease but has the drawback of poor donor site volume. Our mosaic fleur-de-profunda artery perforator flap technique for breast reconstruction has shown to increase volume with the addition of a vertical limb, include full angiosome of perforators, and exhibit donor site morbidity equivalent to a medial thigh lift.

## INTRODUCTION

The expansion in variety of perforator-free flaps in autologous breast reconstruction has provided reconstructive microsurgeons with increased options when the abdomen is not available for autologous reconstruction. Traditional secondary autologous tissue transfer options include latissimus dorsi (LD), superior gluteal artery perforator, inferior gluteal artery perforator, profunda artery perforator (PAP), or transverse upper gracilis (TUG) muscle flap.^1^ A limitation of lower extremity flaps is poor donor site volume. The fleur-de-lis technique has been discussed in a number of different flap locations including: deep inferior epigastric perforator (DIEP), LD, and TUG. This expansion technique, to include both a horizontal and vertical limbs, has shown to increase volume, add vascular territories, and allow for a more favorable breast shape.^2–5^ For those who are not candidates for a DIEP flap and do not desire the unsightly scar of a LD or TUG flap, a PAP scar can be well hidden in the inferior gluteal crease. In addition, in comparison to the TUG, the PAP has shown to have longer pedicle length, larger volume, and does not sacrifice muscle.^6^ Literature review shows how a vertical PAP flap can expand vascular territories and increase donor volume but there remains an absence of discussion regarding the PAP with fleur-de-lis technique.^7^ The mosaic flap, 2 adjacent flaps with separate pedicles from a single vascular source, is advantageous for increasing volume while avoiding addition of separate donor vasculature. Further, it provides enhanced vascularity to avoid potential fat necrosis with the increased harvested volume. The purpose of this case series is to present an innovative surgical technique for breast reconstruction called the fleur-de-PAP, which incorporates mosaic flap properties, and would include both the vertical and horizontal perforator angiosomes.

## CASE DESCRIPTION

### Patient 1

**Fig. 1.**
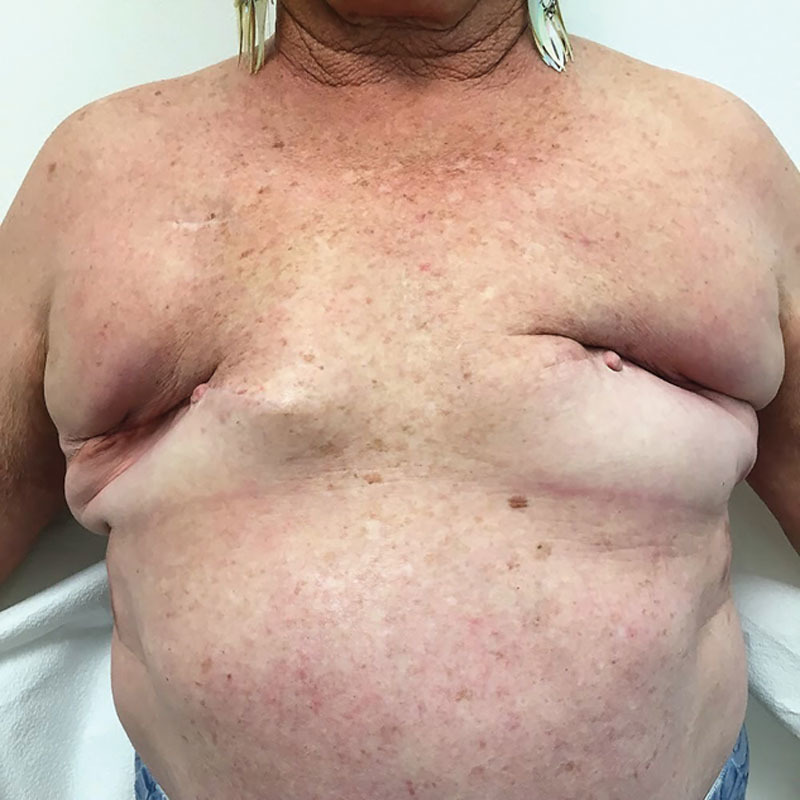
Patient 1: Preoperative photo after bilateral mastectomy and implant reconstruction failure.

A 63-year-old nonsmoking woman, with left breast cancer, opted for bilateral autologous breast reconstruction (Fig. 1). Intraoperatively, the patient was placed in lithotomy position. Skin paddle fleur-de-PAP patterns were drawn on both thighs with a transverse component along the superior-medial thigh, and a vertical component, posterior to the adductor longus. Flaps were elevated with initial incision made over the gracilis muscle anteriorly and dissection performed in a subfascial plane proceeding posteriorly. The dominant perforators through the adductor magnus was identified and circumferentially dissected (Fig. 3). Retrograde dissection was taken to the profunda artery and vein. The vessels were clipped and divided. The superior and posterior incisions were then performed and the flap was harvested, anastomosed to the internal mammary artery and vein. In a similar fashion, the left thigh flap was harvested and taken to the left chest for microvascular anastomosis. The flaps were inset using the transverse portion of the flap to provide superior fullness and the vertical portion of the flap providing an inferior sling along the inframammary fold (IMF). Final flap weights were: right 316 g, left 298 g with each flap measured 22 cm × 7 cm. Perfusion to all components of the skin paddle was confirmed with SPY (Novadaq, Toronto, Ont.) fluorescence imaging.

**Fig. 2.**
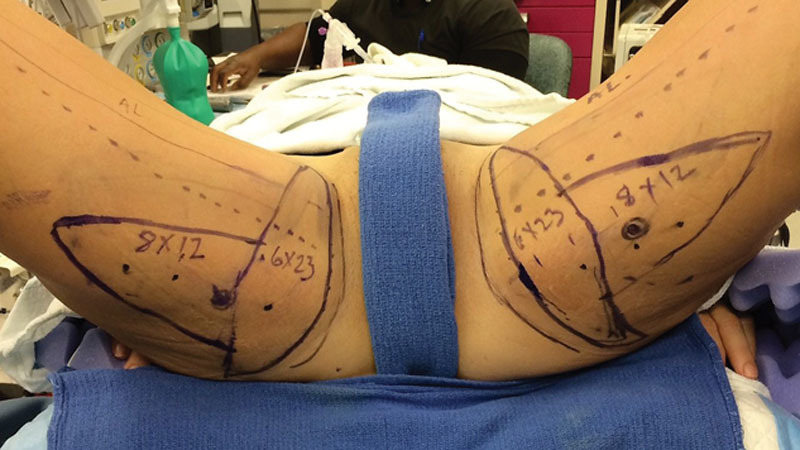
Patient 2: Intraoperative skin markings in the lithotomy position.

At follow-up appointments, the only complication was minimal dehiscence at the T-junction of the thigh incisions bilaterally which was treated with silver nitrate applications. She subsequently underwent second-stage esthetic procedures for contouring by excising the skin paddle, fat grafting for volume in the superior pole (140 mL to right breast and 80 mL to the left breast), and nipple tattooing, without complications (Fig. 4).

**Fig. 3.**
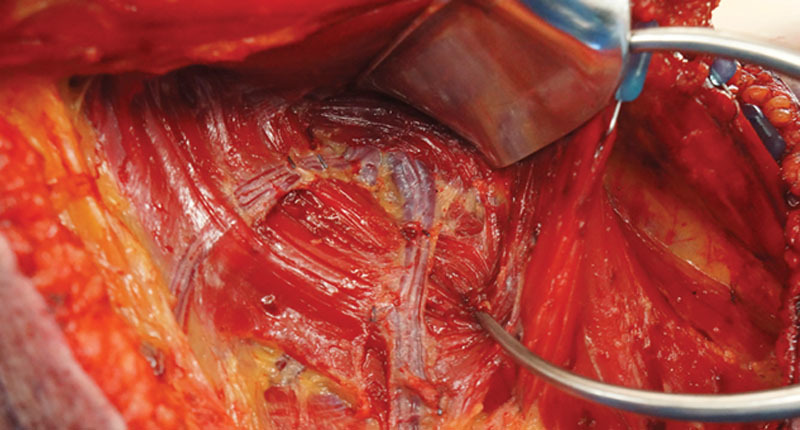
Patient 1: Intraoperative view of 2 profunda artery perforators emerging through adductor magnus, in mosaic branching, to supply both the horizontal and vertical limbs of the flap.

**Fig. 4.**
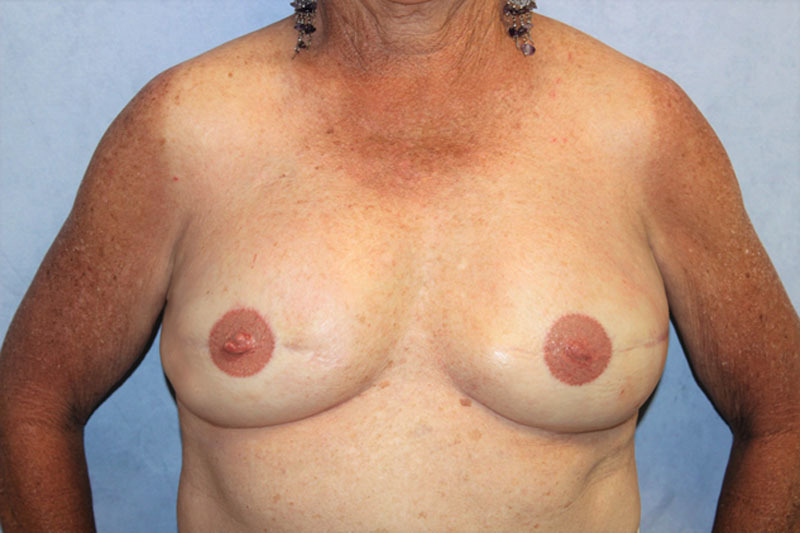
Patient 1: Postoperative results of bilateral breast reconstruction, using fleur-de-PAP free flaps, following second-stage procedure and tattooing at 1.5 years. Not shown is the postoperative thigh views of the donor site demonstrating well-hidden scars in inferior gluteal crease and medial thighs that resemble acceptable medial thigh lift results.

### Patient 2

A 42-year-old woman with unilateral breast cancer who was not a candidate for bilateral DIEP flaps secondary to a previous abdominoplasty. She was offered autologous breast. With the patient in the lithotomy position, bilateral fleur-de-PAP flaps were marked and harvested by means of similar technique as patient 1 (Fig. 2) with flaps weighing 360 and 380 g. The flaps were transferred to the chest wall for microvascular anastomosis using the internal mammary system. Inset was completed using an inverted T-position, with the lateral limbs folded posteriorly to create a 3-dimensional teardrop with greater projection at the inferior pole of the breast.

Her postoperative course was complicated by a surgical-site infection at the left breast, which responded to antibiotics, local debridement, and wound care. A second-stage procedure was completed 6 months later to finalize the reconstruction. At that time, the patient underwent bilateral fat grafting with 150 mL of fat to each breast and had bilateral nipple reconstruction.

## DISCUSSION

The PAP flap has the largest caliber skin paddle (averages of 28 cm × 8 cm) and longest pedicle (average of 10 cm), of all posterior thigh perforators. The PAP has an average of 3 perforators, to supply the medial and posterior thigh skin and subcutaneous tissues, approximately 6 cm below the buttock crease. Computed tomography angiogram scans show that perforators have an equal chance of being medial or lateral to the midline of gluteal crease in vertical distribution.^1,8^ The fleur-de-lis technique allows centering of perforators and ensures inclusion of any variability in perforator outliers by means of the horizontal and vertical limbs. When selecting a breast reconstruction surgery for a patient with limited donor tissue, stacked flaps can provide adequate volume and projection with the sacrifice of multiple donor sites and increased scars.^9^ Modifying the donor site, as with this mosaic fleur-de-lis PAP technique, allows for incorporation of more volume without sacrificing esthetics. One drawback of the extension of this design for skin paddle is the vertical scar. The vertical scar, however, mimics that of a medial thigh lift with minimal appearance from anterior and posterior views.^10^ Both patients exhibited excellent breast esthetic outcome with minimal donor site morbidity. Literature review showed 1 case series for fleur-de-lis gracilis flaps. This series had 17 patients with a 19.3% complication rate for wound dehiscence at the T-junction.^4^

## CONCLUSIONS

When the abdomen is not a viable donor site, the mosaic fleur-de-PAP flap technique for breast reconstruction has shown to increase volume with the addition of a vertical limb, include full angiosome of perforators, and proposed donor site morbidity equivalent to a medial thigh lift.
